# Color-stable highly luminescent sky-blue perovskite light-emitting diodes

**DOI:** 10.1038/s41467-018-05909-8

**Published:** 2018-08-30

**Authors:** Jun Xing, Yongbiao Zhao, Mikhail Askerka, Li Na Quan, Xiwen Gong, Weijie Zhao, Jiaxin Zhao, Hairen Tan, Guankui Long, Liang Gao, Zhenyu Yang, Oleksandr Voznyy, Jiang Tang, Zheng-Hong Lu, Qihua Xiong, Edward H. Sargent

**Affiliations:** 1 Division of Physics and Applied Physics, School of Physical and Mathematical Sciences, Nanyang Technological University, Singapore, 637371 Singapore; 2 Department of Electrical and Computer Engineering, University of Toronto, 10 King’s College Road, Toronto, ON M5S 3G4 Canada; 3 Department of Materials Science and Engineering, University of Toronto, 184 College Street, Toronto, ON M5S 3E4 Canada; 4 Wuhan National Laboratory for Optoelectronics (WNLO) and School of Optical and Electronic Information, Huazhong University of Science and Technology (HUST), 430074 Wuhan, China; 5 NOVITAS, Nanoelectronics Centre of Excellence, School of Electrical and Electronic Engineering, Nanyang Technological University, Singapore, 639798 Singapore; 6 MajuLab, CNRS-UNS-NUS-NTU International Joint Research Unit, UMI-3654, Singapore, 639798 Singapore; 7 Present Address: Shaanxi Institute of Flexible Electronics (SIFE), Northwestern Polytechnical University (NPU), 127 West Youyi Road, 710072 Xi’an, Shaanxi China

## Abstract

Perovskite light-emitting diodes (PeLEDs) have shown excellent performance in the green and near-infrared spectral regions, with high color purity, efficiency, and brightness. In order to shift the emission wavelength to the blue, compositional engineering (anion mixing) and quantum-confinement engineering (reduced-dimensionality) have been employed. Unfortunately, LED emission profiles shift with increasing driving voltages due to either phase separation or the coexistence of multiple crystal domains. Here we report color-stable sky-blue PeLEDs achieved by enhancing the phase monodispersity of quasi-2D perovskite thin films. We selected cation combinations that modulate the crystallization and layer thickness distribution of the domains. The perovskite films show a record photoluminescence quantum yield of 88% at 477 nm. The corresponding PeLEDs exhibit stable sky-blue emission under high operation voltages. A maximum luminance of 2480 cd m^−2^ at 490 nm is achieved, fully one order of magnitude higher than the previous record for quasi-2D blue PeLEDs.

## Abstract (web summary)

Perovskite light-emitting diodes show promising color tunability and device performance but suffer from emission color shift at higher driving voltages. Here Xing et al. report color stable blue light-emitting diodes by drastically increasing the phase purity of the quasi-2D perovskite thin films.

## Introduction

Metal halide perovskites are emerging as a promising candidate for solution-processed optoelectronics, including photovoltaics, photodetectors, lasers and light-emitting diodes (LEDs)^1–18^. Thanks to their excellent properties such as a tunable bandgap, high photoluminescence quantum yield (PLQY), and high color purity, perovskite LEDs (PeLEDs) have attracted intense research interest. Recently, green- and near-infrared-emitting PeLEDs reached impressive external quantum efficiencies (EQEs) exceeding 10%^4,19–21^. However, producing color-stable and efficient blue PeLEDs required for lighting and displays remains challenging, especially in light of limited materials stabilty^22–25^.

Conventional metal halide perovskites exhibit a three-dimensional (3D) structure with a chemical formula of AMX_3_ (A = CH_3_NH_3_ (MA), Cs; M = Pb, Sn; X = Cl, Br, I). The bandgap of the mixed halide perovskites can be tuned monotonically by controlling the halogen component. One widely employed strategy to achieve blue PeLEDs (emission wavelength between 450 and 490 nm) is therefore to employ Cl:Br-mixed halide perovskites as the emissive material. However, the migration of the halogen ions enables phase segregation into Cl-rich and Br-rich phases under illumination and voltage bias^24,26,27^. As a result, the electroluminescence (EL) wavelength undesirably shifts from blue to green during device operation.

**Fig. 1:**
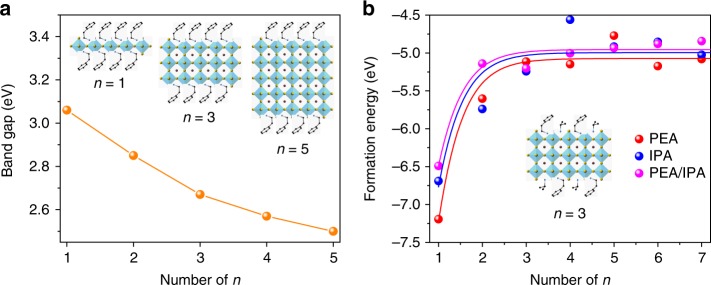
Bandgap and formation energy calculations of quasi-2D perovskite. **a** Bandgap of quasi-2D perovskite with different number of layers, *n*. These values are determined by TA measurement. The inset is the atomic model of the quasi-2D perovskite with *n* = 1, 3, and 5. **b** Calculated formation energy of PEABr, IPABr, and their mixed quasi-2D perovskites with different *n* value. The inset is an atomic model of the PEA/IPA mixed quasi-2D perovskite with *n* = 3

Quasi-two-dimensional (quasi-2D) perovskites provide another pathway to achieve blue PeLEDs. Their reduced-dimensional structure has a standard formula of B_2_A_*n*−1_M_*n*_X_3*n*+1_, where B is a long-chain ligand such as butylammonium (BA) or phenylethylammonium (PEA) (inset of Fig. 1a). By reducing the number of inorganic layers (*n*), one may tune the bandgap of quasi-2D perovskites from ca. 2.6 eV (*n* = 4), to 2.7 eV (*n* = 3), 2.9 eV (*n* = 2), and 3.1 eV (*n* = 1) (Fig. 1a)^28^. Quasi-2D blue PeLEDs have been also reported; however, they exhibit poor color stability under LEDs operation^23,29–32^. This is due to the presence of multiple emission peaks arising from mixed phases that exhibit inefficient charge transfer and different emission features dependent on bias voltage.

So far, PEA-based quasi-2D perovskites exhibited a well-defined single emission peak as a result of efficient charge transfer between quantum wells with various *n* values^8,28,33^. However, PEA-based quasi-2D perovskites that use only Br could not reach blue emission due to the presence of the phase with large *n*^28,33^. We postulate that the formation of different phases within a perovskite film could arise due to the imbalance of crystallization of different phases. Specifically, strong van der Waals interaction between the stacked bulky organic cations^8,28^ makes the formation of 2D perovskite with *n* = 1 most favorable (Supplementary Note 1 and Supplementary Fig. 1). This consumes a major portion of bulk organic cations and leaves the short-chain precursors (those associated with the repeat unit of 3D crystals) to nucleate phases with high *n*. As a result, a mixed phase that includes high-*n* components shows green emission due to efficient carrier funneling to the smallest-bandgap phase.

In this report, we therefore aim at slowing the formation of the pure *n* = 1 phase to improve the monodispersity of *n* = 2, 3, 4 phases enabling more controlled formation of a quasi-2D PEA_2_A_*n*−1_Pb_*n*_X_3*n*+1_ perovskite with a desired *n*. We achieve this by replacing long ligands (PEA) with shorter ones (iso-propylammonium, IPA), an improvement that reduces van der Waals interactions, therefore destabilizing the *n* = 1 phase. Slower crystallization of *n* = 1 enhances purity and monodispersity of *n* = 2, 3, and 4 phases. The as-synthesized perovskite films exhibit a single emission peak and color-stable blue emission (477 nm) with a record PLQY of 88%. The PL wavelength exhibits no shift under 325-nm irradiation (7 W cm^−2^) for 1 h. We further fabricate sky-blue PeLEDs with a maximum luminance of 2480 cd m^−2^ at the wavelength of 490 nm, a luminance that is one order of magnitude higher than the previous record for quasi-2D blue PeLEDs (Supplementary Table 1)^23,29–32^.

## Results

### Synthesis and structural analysis of perovskite films

We synthesized blue emission perovskites by mixing PbBr_2_, MABr, CsBr, PEABr, and IPABr in the solution of dimethyl sulfoxide (DMSO) and using a one-step spin-coating method (see Methods). The mixture of MA and Cs cations has been shown previously to improve the crystallinity and stability of perovskite films^34^. The ratio of MABr/CsBr to PEABr and PbBr_2_ in the precursor solution was kept constant at 3:4:5, targeting an average perovskite formula PEA_2_A_1.5_Pb_2.5_Br_8.5_ (A = MA and Cs). We explored a number of additives to tune the emission properties: IPABr, ethylammonium bromide (EABr), *n*-propylammonium bromide (PABr), and butylammonium bromide (BABr). We found that only the addition of IPABr resulted in a perovskite product with high PLQY.

**Table 1:** Summary of the composition of the perovskite samples and their corresponding PL peaks and PLQY

	CsPbBr_3_	MAPbBr_3_	PEA_2_PbBr_4_	IPABr	PL peak (nm)	PLQY%
Sample I	0.5	0.1	0.4	0	504	69
Sample II	0.5	0.1	0.4	0.1	498	79
Sample III	0.5	0.1	0.4	0.2	491	83
Sample IV	0.5	0.1	0.4	0.4	480	73
Sample V	0.45	0.15	0.4	0.4	477	88
Sample VI	0.4	0.2	0.4	0.4	473	82
Sample VII	0.5	0.1	0.4	0.6	467	53

The ratio of the precursors is mole ratio. Sample I is the control sample

Density functional theory (DFT) simulations (Fig. 1b) show that when PEABr and IPABr ligands are used in combination, the formation energy of the 2D perovskite with *n* = 1 changes from −7.2 (more stable) to −6.5 eV (less stable), indicating a partial destabilization of *n* = 1 phase compared to the *n* = 2, 3, and 4 phases. Due to stoichiometry constraints, we obtained only quasi-2D perovskites with low number of inorganic layers, which is critical to achieving blue emission.

X-ray diffraction (XRD) results reveal the layered structure of formed perovskite thin films (Supplementary Fig. 2). Diffraction peaks at 4.35, 8.74, 13.5, 27.2, and 31.55 degrees correspond to the (0 0 *l*) series of Bragg reflections of the quasi-2D perovskite. The lowest angle of 4.35° corresponds to the lattice fringe of 2.0 nm, which we assign to the interlayer spacing in the quasi-2D perovskite phase with *n* = 2^35^.

### Optical properties of perovskite films

**Fig. 2:**
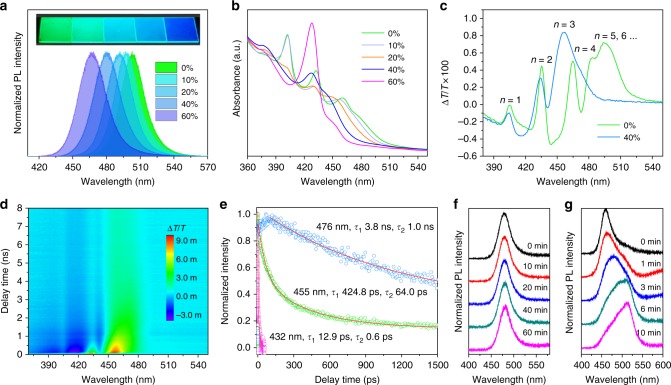
Photophysical properties of quasi-2D perovskite. **a** PL and **b** absorption spectra of perovskite PEA_2_A_1.5_Pb_2.5_Br_8.5_ with 0−60% IPABr additive. The inset is a photograph of the corresponding films under 365 nm UV irradiation. **c** TA spectra of PEA_2_A_1.5_Pb_2.5_Br_8.5_ with 0 and 40% IPABr. **d**, **e** TA time delay of PEA_2_A_1.5_Pb_2.5_Br_8.5_ with 40% IPABr. PL spectra of quasi-2D perovskite (**f**) and MAPbCl_1.5_Br_1.5_ (**g**) film under continuous laser radiation (325 nm, 7 W cm^−2^) for different exposure times

Figure 2a shows PL spectra of the perovskite films. Pristine PEA_2_A_1.5_Pb_2.5_Br_8.5_ perovskite emits at 504 nm; the addition of IPABr progressively shifts the PL peak from 497 to 467 nm as the IPABr/Pb ratio increases from 10 to 60%. The corresponding photograph of the perovskite films under UV light is shown in the inset of Fig. 2a. The highest PLQY of 73% was obtained with an IPABr ratio of 40% (Table 1). By tuning the fractions of MABr and CsBr, we further optimized the PLQY and obtained a highest value of 88% with the PL peak at 477 nm.

UV−Vis absorption spectra were used to characterize the optical bandgap and excitonic features of these perovskites. In Fig. 2b, multiple excitonic absorption features at 3.1, 2.9, 2.7, and 2.6 eV correspond to the perovskite phases with *n* = 1, 2, 3, and 4, respectively^28^. We also observed a blueshift of the absorption edge of the perovskite films with increased IPABr fraction. We attribute this blueshift to the fact that the component of lowest-*n* and highest-*n* phases was inhibited gradually, while the intermediate *n* phase (*n* = 2, 3, 4) grew faster instead (Fig. 2b).

### Transient absorption spectroscopy

To gain further insights into the dynamics of photocarriers in the bright blue quasi-2D perovskites, we carried out transient absorption (TA) studies (Fig. 2c). In the control sample (without IPABr), we observed five distinct bleach peaks at 405, 435, 465, 483, and 495 nm. The peak locations of these transitions are in good agreement with peaks in the steady-state absorption spectra (Fig. 2b). These results show that the quasi-2D perovskite PEA_2_A_1.5_Pb_2.5_Br_8.5_ films are not single-phase, but instead consist of a mixture of phases with *n* = 1, 2, 3, 4, 5, and higher^28^. The band edge energies of the perovskites with *n* ≥ 5 are closely spaced and difficult to distinguish. Therefore, we propose that bulk perovskite phases with larger *n* values may also exist in the PEA_2_A_1.5_Pb_2.5_Br_8.5_ films. In contrast, in the sample with 40% IPABr additive, only four distinctive phases with *n* = 1, 2, 3, and 4 were observed with no spectral or XRD features that are normally attributed to *n* ≥ 5.

We then investigated the decay kinetics of multi-exciton bleaching (Fig. 2d). We extracted three distinct kinetics at 432, 455, and 476 nm corresponding to *n* = 2, 3, and 4, that revealed the following time constants after fitting (Fig. 2e):

13 ± 0.1 ps and 0.6 ± 0.01 ps (432 nm);

420 ± 10 ps and 64 ± 2 ps (455 nm); and

4 ± 1 ns and 1.0 ± 0.1 ns (476 nm)

In light of previous reports of very fast charge transfer among perovskite phases^36^, we assign the fast component at 432, 455 nm (*n* = 2, 3) to the charge transfer to perovskite with *n* = 4 and ascribe the slow component to charge trapping^28^. We attribute the fast and the slow features at 476 nm (emitting domain, *n* = 4) to a nonradiative and a radiative process, respectively. Notably, at 476 nm, the timescale of the rise of the bleach intensity ∆*T*/*T* (during the first 100 ps) is in good agreement with the charge transfer (reflected by lifetime decay) that was previously reported for a perovskite phase with *n* *=* 3^36^.

We further studied the color stability of these improved quasi-2D perovskites. The PL spectra of the PEA_2_A_1.5_Pb_2.5_Br_8.5_ perovskite with 40% IPABr under continuous 325 nm laser radiation (7 W cm^−2^) are shown in Fig. 2f. The PL maximum is unchanged even after irradiation for 60 min. In contrast, the PL peak of a mixed halide perovskite MAPbBr_1.5_Cl_1.5_ film shifts from 460 to 515 nm within 10 min of illumination under the same conditions (Fig. 2g).

### Device structure and performance

**Fig. 3:**
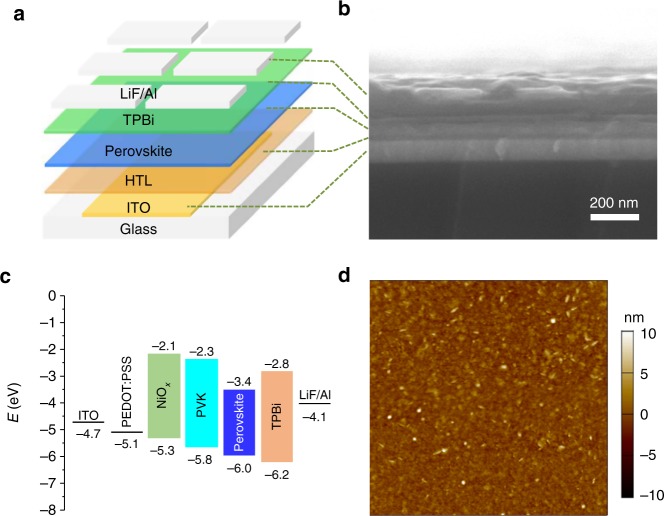
Device structure of PeLEDs. **a** Device structure, **b** cross-sectional SEM image and **c** energy level alignment of the PeLEDs, showing conduction and valence-band levels with respect to vacuum. The energy level of perovskite layer was determined by UPS measurement. **d** AFM image of perovskite film on PEDOT:PSS. Scan area is 5 × 5 μm^2^. The surface roughness was measured to be around 1.0 nm

Encouraged by the above results, we further fabricated PeLEDs using perovskite films with highest PLQYs (Sample V, see Methods for details). As indicated in Fig. 3a, b, we began with the following PeLED architecture: ITO/PEDOT:PSS (30 nm)/perovskite film (40–125 nm)/TPBi (50 nm)/LiF (1 nm)/Al (100 nm). We measured the energy levels of the perovskites by using ultraviolet photoelectron spectroscopy (UPS, Supplementary Fig. 3). As shown in the atomic force microscopy (AFM, Fig. 3d) image, the surface roughness of the perovskite film on PEDOT:PSS is only 1 nm, which is sufficiently small to avoid current leakage.

**Fig. 4:**
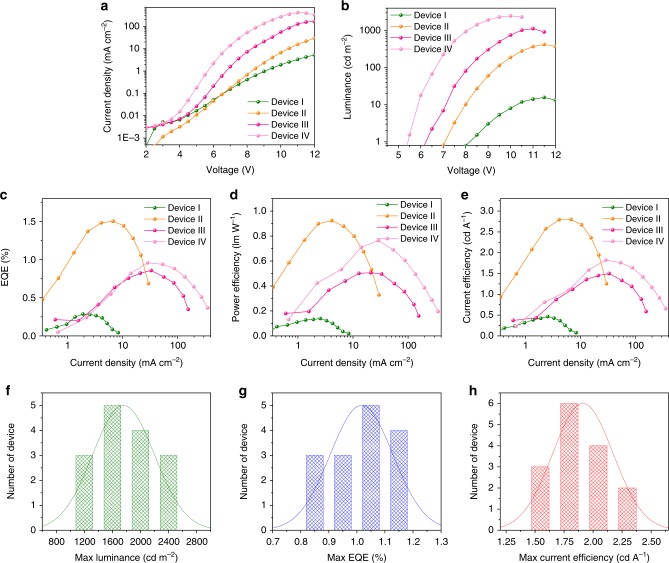
EL performance of PeLEDs. **a** Current density-voltage, **b** luminance-voltage, **c** EQE-current density, **d** power efficiency-voltage and **e** current efficiency-voltage characteristics of PeLEDs made from perovskite films with different thickness: Device I (125 nm), Device II (80 nm), Device III (60 nm), and Device IV (40 nm). **f**−**h** Histogram of maximum luminance, EQEs, current efficiency for 15 Devices IV from four batches. Average luminance of 1758 cd m^−2^ with a relative standard deviation of 22%, average EQE of 1.0% with a relative standard deviation of 11% and average current efficiency of 1.9 cd A^−1^ with a relative standard deviation of 14%

Compared to 3D perovskites, quasi-2D perovskites exhibit lower conductivity and thus their LED architecture usually requires thinner active layers. To explore the effect of the thickness on our device performance, we studied the PeLEDs with thicknesses of the active layer ranging from 125 nm (Device I) to 80 nm (Device II), 60 nm (Device III), and 40 nm (Device IV) (Supplementary Fig. 4). We found that devices with thinner active layers showed both increased current density and luminance (Fig. 4a). We also found that higher brightness (2480 cd m^−2^, Device IV, Fig. 4b) was achieved through a systematic reduction of thickness. The best EQEs, power efficiency, and current efficiency of 1.5%, 0.92 lm W^−1^, and 2.8 cd A^−1^, respectively, were found in Device II (Fig. 4c−e). We propose that a higher EQE can be achieved through improvement of the electron (ETLs) and hole (HTLs) transport layers to optimize the charge carrier injection and blocking functions. As indicated in Fig. 4b, c, the maximum luminance and the maximum EQE were obtained from different devices. Luminance (*L*) is proportional to the product of current density (*J*) and efficiency (EQE), indicating that luminance is affected by both current density and efficiency. While thinner perovskite films achieve higher *J*, they do not always have higher EQE. EQE is governed by exciton and charge carrier dynamics. Most exciton quenching processes scale with charge carrier concentrations, making quenching processes most pronounced at high current densities. The reduction of perovskite thickness also increases the chance of charge carrier leakage. More exciton quenching and more charge carrier leakage will result in lower EQE. Therefore, increasing the maximum current density can compensate for some of these EQE losses; however, the best luminance could still be realized in a device with higher maximum current density. To characterize the reproducibility of the PeLED, we summarized the sample results such as maximum luminance, EQE, and current efficiency of Devices IV with record luminance. Figure 4f−h shows an average luminance of 1758 cd m^−2^ with a relative standard deviation of 22%, an average EQE of 1.0% with a relative standard deviation of 11% and an average current efficiency of 1.9 cd A^−1^ with a relative standard deviation of 14%.

**Fig. 5:**
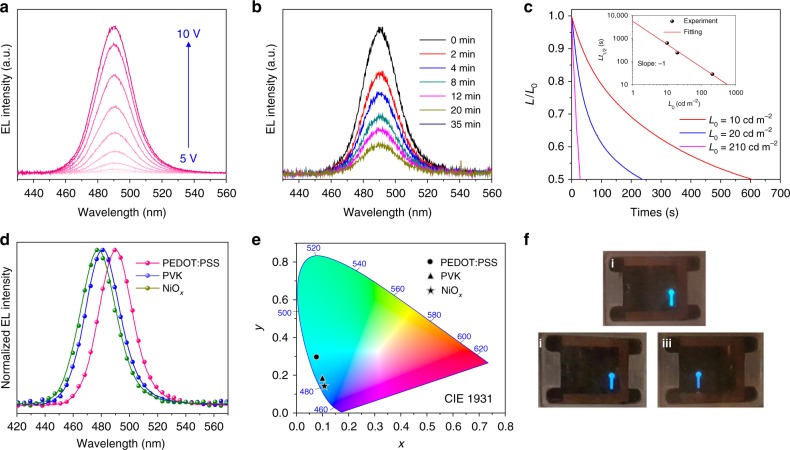
EL spectra of PeLEDs. **a** EL spectra of PeLEDs operating under different voltage. **b** EL spectra of PeLED operating with various exposure times. **c** Lifetime measurement of the PeLEDs device at different initial luminance. The inset is estimated half-lifetime using the stretched exponential decay. **d** EL spectra and **e** CIE 1931 chromatic coordinates of the PeLEDs with PEDOT:PSS, PVK or NiO_*x*_ as HTL. **f** Digital photographs of the operating PeLEDs with PEDOT:PSS (i), PVK (ii), and NiO_*x*_ (iii) as the HTL materials, respectively

The color stability of the PeLEDs was tested during the device operation. As bias voltage increased from 5 to 10 V, the EL intensity increased correspondingly, and the EL peak of 490 nm and full-width at half-maximum (FWHM) of 28 nm remained stable (Fig. 5a). When we further gradually increased bias voltage up 13 V, the EL intensity decreased accordingly mainly due to the degradation of the perovskite materials (Supplementary Fig. 5), yet both the EL peak position and FWHM remained unchanged. The EL spectra of the PeLEDs also changed negligibly under continuous operation for 35 min (Fig. 5b). The stability of PeLEDs is still a big challenge, especially for blue PeLEDs, which work under higher driving voltage compared to green and red PeLEDs. The sky-blue PeLEDs lifetimes were measured to be 10, 4, and 0.5 min at initial luminance of 10, 20, and 210 cd m^−2^, respectively (Fig. 5c). This is consistent with the empirical scaling law $L_0^n \cdot T_{1/2}\ {{ = C}}$, where *L*_0_ is initial luminance, *T*_1/2_ is LEDs half-lifetime, *n* is a scaling factor and *C* is a constant. This equation implies that higher initial luminance will result in lower lifetime.

To investigate the effect of the additive IPABr on LEDs performance, we fabricated LEDs based on perovskite samples I, II, III, IV, and VII with the thickness of 40 nm. The current densities of these devices decreased as the fraction of IPABr increased; meanwhile, their maximum luminance decreased from ca. 33,000 (Sample I device) to 32,500 (Sample II device), 21,000 (Sample III device), 1700 (Sample IV device) and 2 cd m^−2^ (Sample VII device) accordingly (Supplementary Fig. 6). This may be due to the fact that low conductive ligands blocking charge injection. The maximum EQE of Sample I and II devices are similar (ca. 2.6%), and this value increases to 4.0% in Sample III device, and then decrease to 2.2% in Sample IV device and further to 0.05% in Sample VII device. This is consistent with the trend of PLQY. The EL signal of these devices gradually blue shifted from 513 to 474 nm as the fraction of IPABr increased from 0 to 60%. The EL peaks also red shifted compared to their corresponding PL peaks.

The EL redshift of PEDOT:PSS-based PeLEDs may be induced by the ionic nature of PEDOT:PSS. We also therefore explored devices with neutral HTLs PVK and NiO_*x*_. Using PVK and NiO_*x*_ improved hole injection, which can be explained by their lower HOMO level compared to that of PEDOT:PSS (Fig. 3c). The threshold to achieve 1 cd m^−2^ decreased from 6.2 V (PEDOT:PSS) to 3.8 V (PVK) and 4.7 V (NiO_*x*_) (Supplementary Fig. 7). Their corresponding EL spectra are consistent with the PL spectra of the perovskite film on glass substrate, located at 481 nm (PVK) and 477 nm (NiO_*x*_) (Fig. 5d). These enable CIE 1931 chromatic coordinates (0.098, 0.174) and (0.107, 0.137) for purer-blue devices (Fig. 5e). Figure 5f shows the photographs of operating PeLEDs with PEDOT:PSS, PVK and NiO_*x*_ as the HTL material, respectively. Emission color stability was also monitored during device operation. Even though the operating voltage was increased to 8.5 V, the EL intensity increased with the EL peak positions and FWHM remaining stable (Supplementary Fig. 8).

To identify the influence of different HTLs on the structure of the perovskite films, we first studied the PL spectra of the perovskite films fabricated on glass, NiO_*x*_, PVK, and PEDOT:PSS substrates, respectively. The PL peaks of the perovskite films on glass and NiO_*x*_ are all located at 477 nm; for PVK substrate, the PL peak slightly redshifts to 478 nm; however, the PL peak redshifts to 488 nm for PEDOT:PSS substrate (Supplementary Fig. 9). UV−Vis absorption and TA spectra were used to characterize the bandgap and excitonic feature of these perovskite films (Supplementary Fig. 9). We also observed a redshift by 10 nm of the absorption edge of the perovskite film on PEDOT:PSS substrate. In TA spectra, three distinct bleach peaks were observed from perovskite films on glass, NiO_*x*_, and PVK substrates, which correspond to the perovskite phases with *n* = 2, 3, and 4. In contrast, in the perovskite film on PEDOT:PSS substrate, the perovskite phase with *n* > 4 appears, as evidenced from one additional feature around 480–490 nm. Therefore, the EL redshift of the LED device with PEDOT:PSS as HTL is attributed to the perovskite phase with large *n* induced by PEDOT:PSS layer.

## Discussion

In this study we report the synthesis of color-stable blue-emitting perovskite thin film with high PLQYs. Our approach was to deliberately select cation combinations to modulate the crystallization of quasi-2D perovskites. When IPA and PEA ligands were used simultaneously, the growth of lowest-*n* and highest-*n* phases was inhibited, allowing the growth of intermediate-*n* phases to dominate instead. The resultant films display highly efficient PL and stable blue emission. Efficient and color-stable sky-blue PeLEDs have been made from these perovskite films and the devices show record high bright luminance and high EQE.

## Methods

### Materials and chemicals

All alkylamine bromides were purchased from Dyesol. Cesium bromide (CsBr) and lead bromide (PbBr_2_) were purchased from Alfa Aesar. DMSO, toluene, chlorobenzene, and lithium fluoride (LiF) were purchased from Sigma-Aldrich. PEDOT:PSS (AI 4083) was purchased from Heraeus. 1, 3, 5-tris(*N*-phenylbenzimiazole-2-yl)benzene (TPBi) were purchased from Lumtec. All chemicals were used as received.

### Perovskite film fabrication

The precursor solution was prepared by dissolving certain quantities of PbBr_2_, CsBr, MABr, PEABr, and IPABr in DMSO under continuous stirring for 2 h at room temperature. The resulting solution was spin-coated onto the substrate via a one-step process 5000 r.p.m. for 60 s. During the spin step, 200 μL of toluene were poured onto the substrate. The resulting films were then annealed at 70 °C for 10 min to remove the residual solvent. The thickness of the perovskite film was tuned by using the precursor solution with different Pb concentrations 0.5, 0.3, 0.2, and 0.15 M. Their corresponding thicknesses are around 125, 80, 60, and 40 nm, respectively.

### Perovskite film characterizations

The structure of as-grown samples was characterized by using Panalytical X’Pert Pro diffractometer. UV−Vis absorption was measured by using LAMBDA 950 UV/Vis/NIR spectrometer. The morphology of the perovskite films was characterized by AFM (Cypher ES SPM) in the AC mode and field-emission scanning electron microscopy (FE-SEM, JEOL JSM-7001F). UPS spectra of the perovskite films were measured on ITO-coated glass substrate. UPS was performed in a PHI5500 Multi-Technique system using nonmonochromatized He−Iα radiation (*hν* = 21.22 eV). All work functions and valence-band measurements were performed at a takeoff angle of 88°, with chamber pressure near 10^−9^ Torr.

### PL/PLQY measurements

Steady-state PL was recorded using a Horiba Fluorolog system equipped with a single grating and a monochromatized Xe lamp was used as the excitation source. PLQY measurements were done by coupling a Quanta-Phi integrating sphere to the Horiba Fluorolog system with optical fiber bundles. Both excitation and emission spectra were collected for the two cases of the sample directly illuminated by the excitation beam path in the integrating sphere and the empty sphere itself. The monochromatized Xe lamp was used as excitation source with wavelength of 400 nm and power of 1 mW cm^−2^. PL stability was measured on HR800 Raman spectrograph and 325 nm laser was used as excitation source.

### Ultrafast transient absorption spectroscopy

The 800 nm output pulse laser (1 KHz repetition rate, 100 fs pulse width) from a commercial Ti:Sapphire regenerative amplifier (Spectra-Physics Spitfire) is split into two paths. One beam goes through a mechanical delay stage to pump a sapphire/CaF_2_ crystal to generate a light continuum to serve as the probe pulse. The second beam is sent to an optical parametric amplifier (Spectra-Physics TOPAS) to generate pump pulses (380 nm, 2.8 μW). The pump and probe pulses with a cross-polarization configuration are collinearly focused on samples with a beam size of 100 and 50 μm, respectively, by using parabolic mirrors. A mechanical chopper with a synchronized readout of a CMOS detector is used for acquisitions of probe spectra with and without pump-induced changes, enabling calculation of a relative differential transmission. The spectra resolution is 1~2 nm across the detecting range.

### First-principle calculations

We used ground state DFT with the Perdew−Burke−Ernzerhof (PBE)^37^ GGA exchange correlation functional as implemented in the Vienna ab initio simulation package (VASP)^38^ to perform structural optimization of the perovskites. All calculations allowed for spin polarization. We used plane wave energy cut-off of 450 eV and Gaussian smearing (0.2 eV) to converge the electronic problem. The Monkhorst-Pack *k*-points mesh of 900 per atom and the force convergence criterion of 0.0005 eV per atom × *N* atoms in the unit cell were used as implemented in the MPRelaxSet class of the Pymatgen Python package^39–42^. The formation energy of the perovskites was calculated according to the following formula:

$$\begin{array}{ll}{\mathrm {FE}} = E(L_2{\mathrm {MA}}_{n - 1}{\mathrm {Pb}}_n{\mathrm {Br}}_{3n + 1}) - 2E(L{\mathrm {Br}})\\ \,\,\,\,\,\,\,\,\,- (n - 1)E({\mathrm {MABr}}) - nE({\mathrm {PbBr}}_2)\\ \!\!\!\!\!\!\!\!\!\!\!\!\!\!\!\!\!\!\!\!\!\!\!\!\!\!\!\!\!\!\!\!\!\!\!\!\!\!\!\!\!\!\!\!\left( {L = {\mathrm{MA,}}\,{\mathrm{PEA,}}\,{\mathrm{IPA}}} \right)\end{array}.$$

### PeLEDs fabrication

Thin films of PEDOT:PSS, PVK, and NiO_*x*_ were prepared using spin-coating of the precursor solution onto the prewashed patterned ITO-glass substrates. PEDOT:PSS (Clevios PVP AI 4083) solution was spin-coated in air at 4000 r.p.m. for 60 s followed by annealing process in air at 150 °C for 30 min. For the deposition of NiO_*x*_ layer, 0.5 M nickel formate dihydrate ethylene glycol solution containing 1 molar equivalents of ethylenediamine was filtered with 0.45 μm nylon filter and spin-coated at 4000 r.p.m. for 90 s followed by annealing process at 300 °C in air for 60 min. After the substrates were cooled down to room temperature, O_2_ plasma was used to treat the NiO_*x*_ films at a power of 50 W for 5 min. Substrates were then used for device fabrication immediately. For the deposition of PVK layer, PVK/chlorobenzene (5 mg mL^−1^) solution was spin-coated on the ITO-glass substrate at 3000 r.p.m. for 60 s, and annealed at 150 °C for 30 min in a nitrogen-filled glovebox.

Perovskite precursor solutions were spin-coated on the PEDOT:PSS film as described above. TPBi (50 nm) and LiF/Al electrodes (1 nm/100 nm) were deposited using a thermal evaporation system through a shadow mask under a high vacuum of less than 10^−4^ Pa. The device active area was 6.4 mm^2^ as defined by the overlapping area of the ITO and Al electrodes.

### PeLEDs characterizations

The luminance−current density−voltage characteristics were collected by using a HP4140B picoammeter. The absolute EL power spectra of the devices were collected using an integrating sphere and an Ocean Optics USB4000 spectrometer by the mounting of the devices on the wall of the integrating sphere. The EQEs were then calculated through the measured absolute power spectra and the current density. The lifetime of LEDs was measured at constant current density. In total, about 200 PeLEDs were fabricated and tested in this work.

## Electronic supplementary material

Supplementary Information

## Data Availability

The data that support the findings of this study are available from the corresponding authors upon reasonable request.
