# Supplementary material for: Color-stable highly luminescent sky-blue perovskite light-emitting diodes
Source: Nat Commun. 2018 Aug 30;9:3541. doi: 10.1038/s41467-018-05909-8 (PMC6117319; doi:10.1038/s41467-018-05909-8)
Supplement: Supplementary file 1 — Supplementary Information [file 41467_2018_5909_MOESM1_ESM.pdf]

Supplementary Information

Color-Stable Highly Luminescent Sky-Blue Perovskite Light-Emitting Diodes

Xing *et al.*

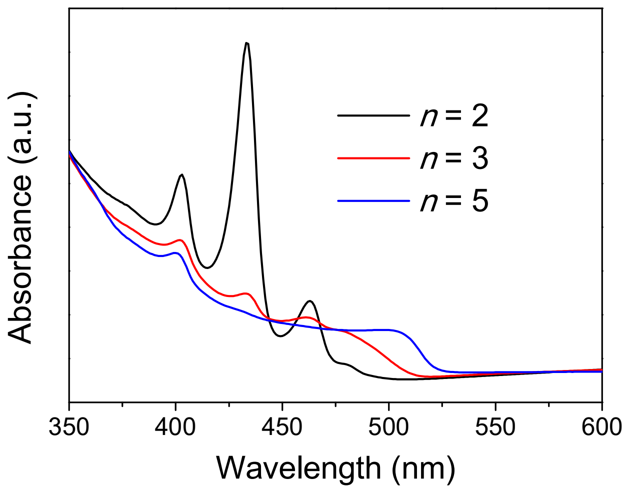

Supplementary Figure 1. Absorption spectra of perovskite films fabricated from $PEA_2Cs_{n-1}Pb_nBr_{3n+1}$ precursor solution with n = 2, 3 and 5.

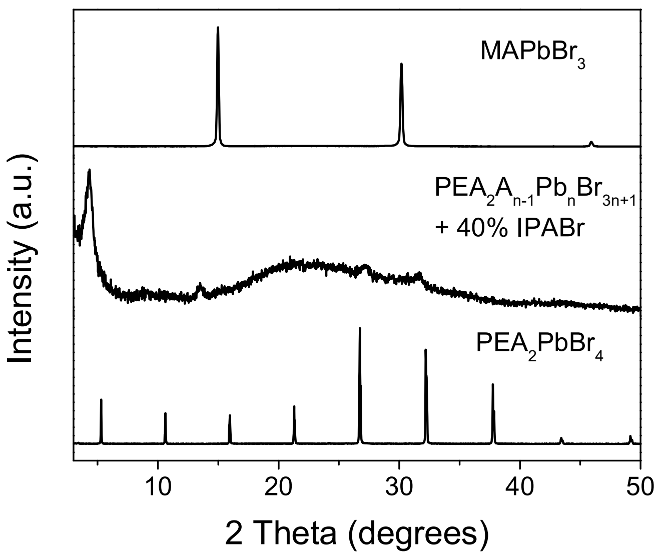

Supplementary Figure 2. XRD patterns of samples $MAPbBr_3$, PEA_2PbBr_4 and $PEA_2A_{n-1}Pb_nBr_{3n+1}$ with 40% IPABr additive.

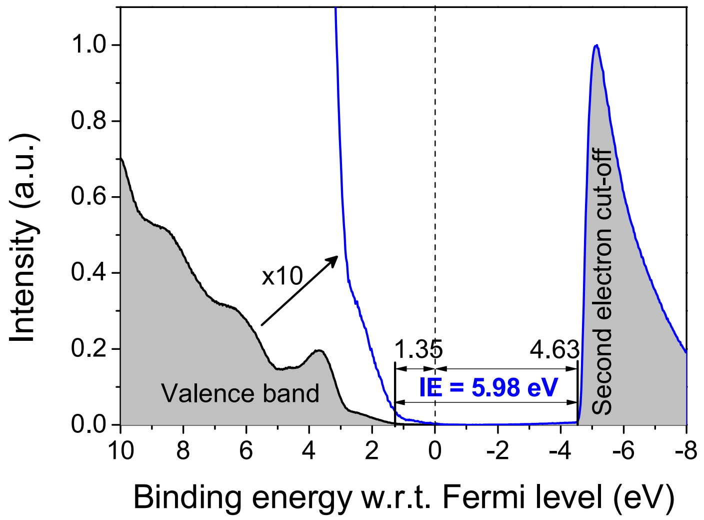

Supplementary Figure 3. Valence band maximum (VBM) and work function (WF) of perovskite film measured via UPS measurement. The VBM of -6.0 eV and CBM of -3.4 eV were obtained by considering band edge of UV-Vis absorption spectra.

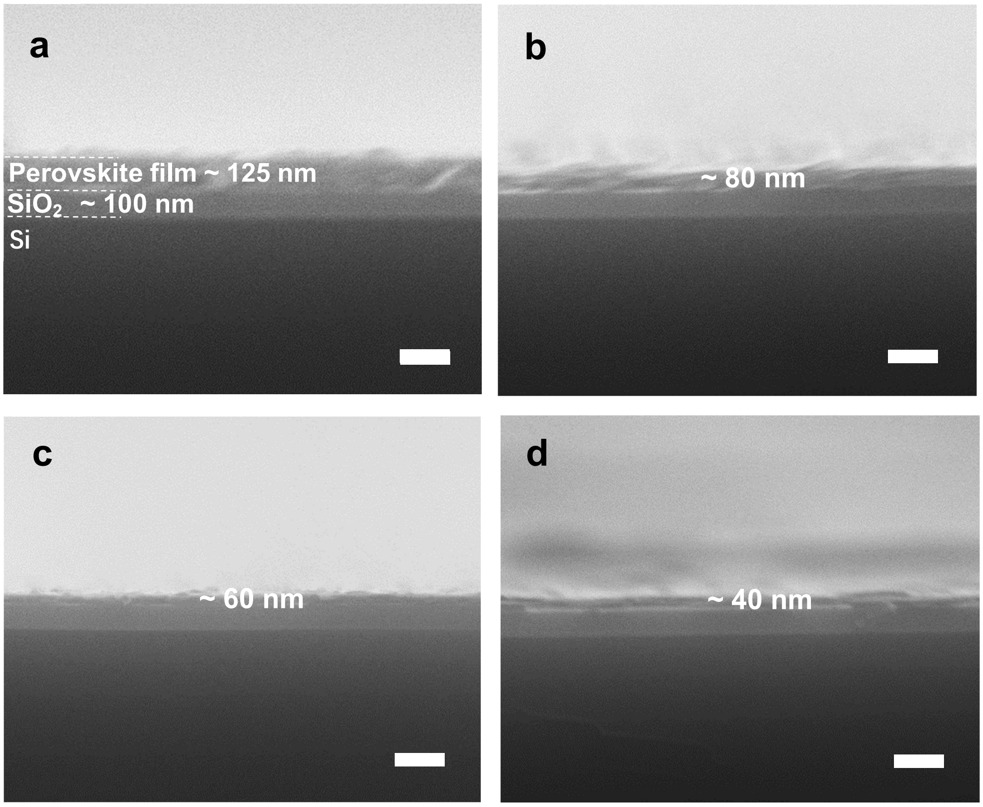

Supplementary Figure 4. Cross-sectional SEM images of perovskite films on silicon with 100 nm SiO_2, which were made from the precursor solution with concentration (a) 0.5 M, (b) 0.3 M, (c) 0.2 M, (d) 0.15 M (scale bar: 200 nm). The thickness of the perovskite film on silicon is same as that on PEDOT:PSS. The thicknesses of the perovskite films are 125, 80, 60 and 40 nm, respectively.

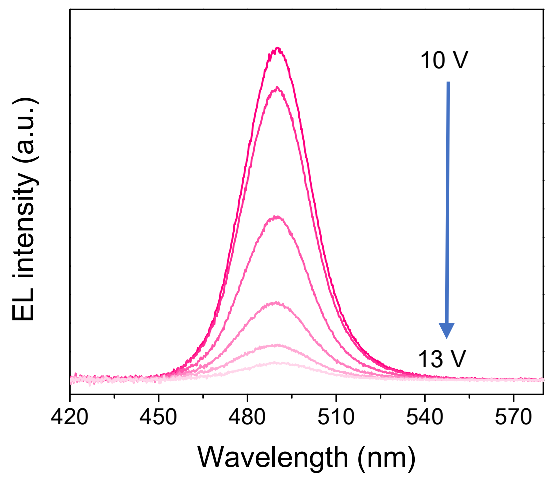

Supplementary Figure 5. EL spectra of PeLEDs operating under the voltage from 10 to 13V.

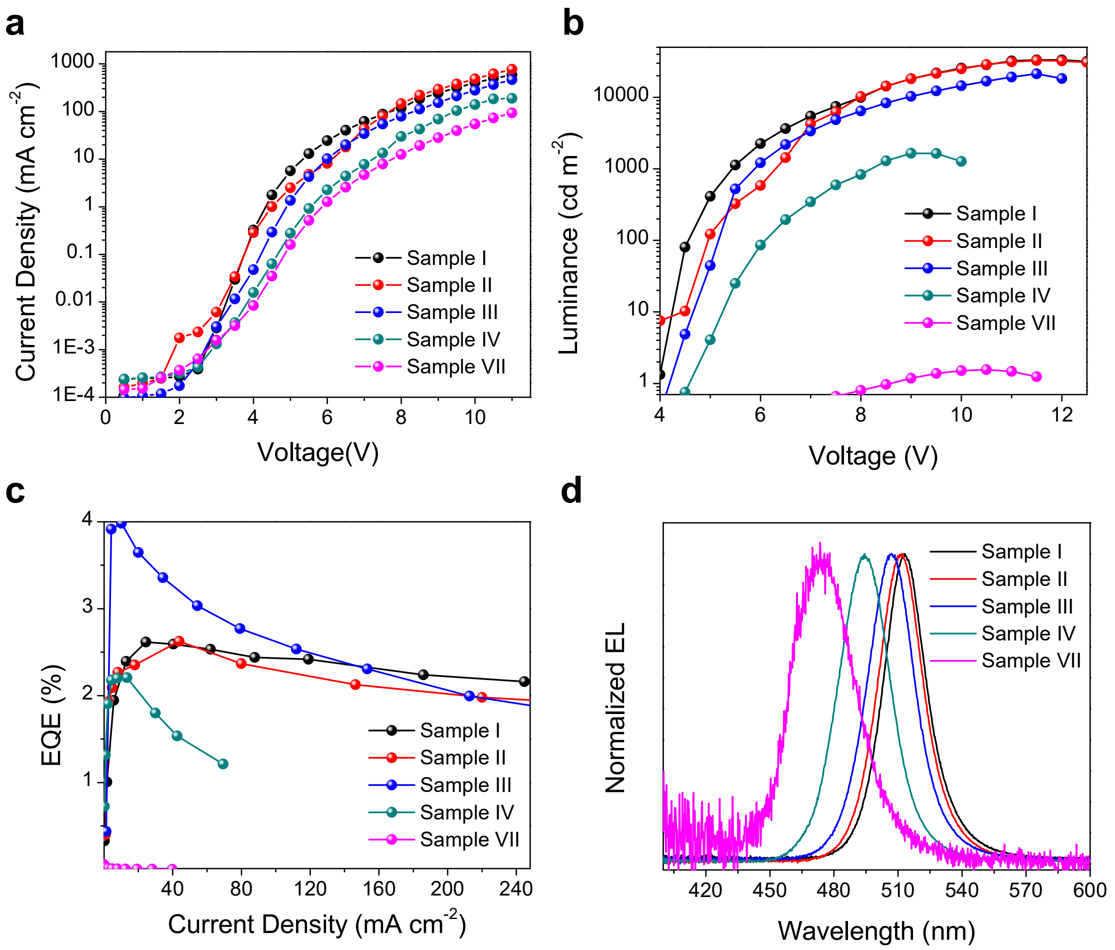

Supplementary Figure 6. (a) Current Density-Voltage, (b) Luminance-Voltage, (c) EQE-Current Density and (d) EL spectra characteristics of the PeLEDs made from Samples I, II, III, IV and VII with thickness of 40 nm. The corresponding EL peaks are located at 513, 511, 507, 494 and 474 nm, respectively.

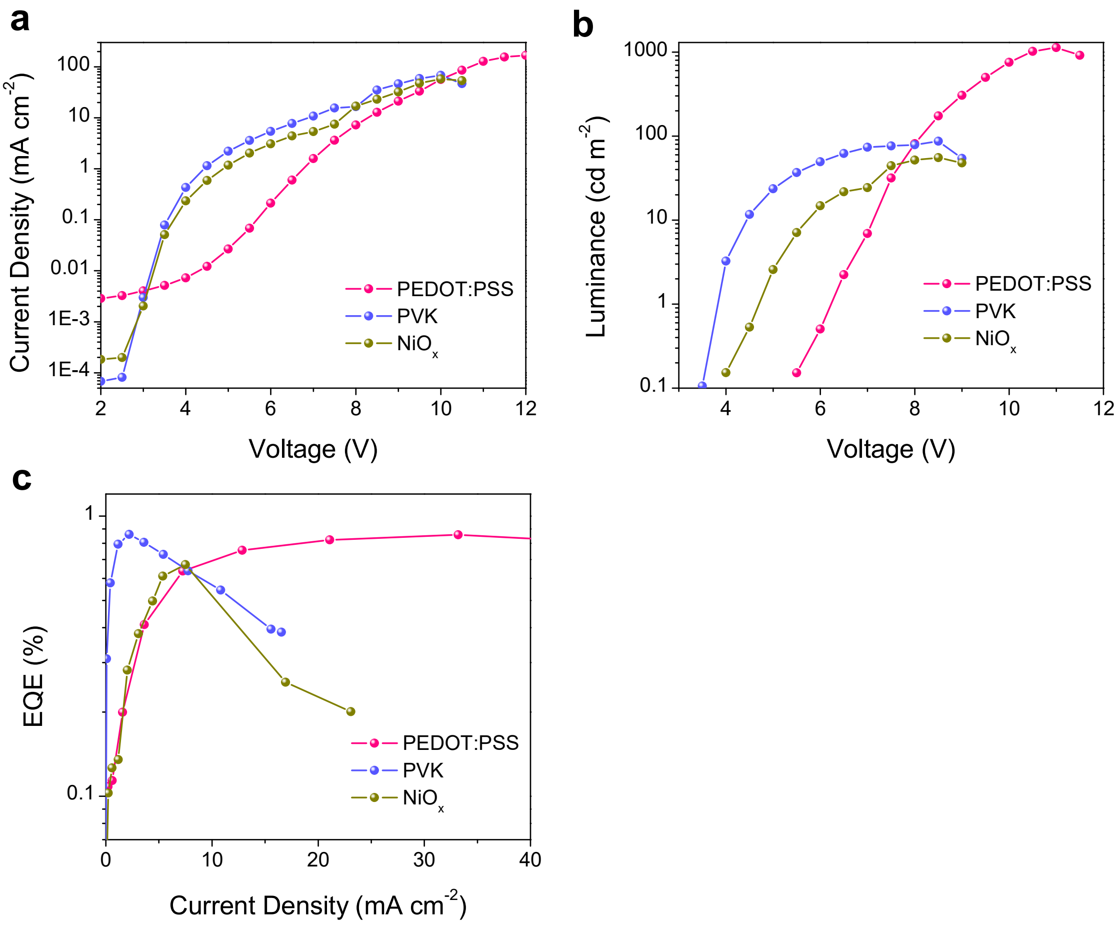

Supplementary Figure 7. (a) Current Density-Voltage (b) Luminance-Voltage, (b) EQE-Current Density characteristics of the PeLEDs with PEDOT:PSS, PVK and NiO_x as the HTL material.

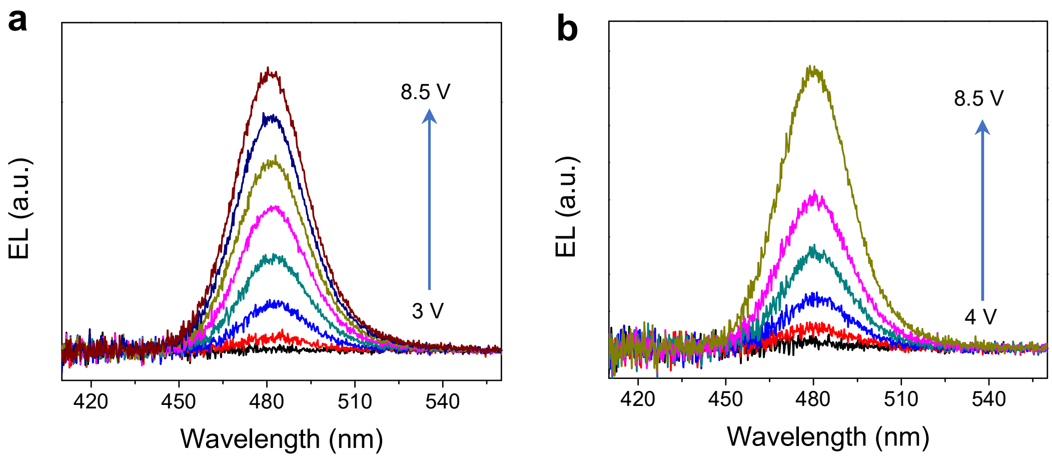

Supplementary Figure 8. EL spectra measured at various operational voltages of the PeLEDs with (a) PVK or (b) NiO_x as the HTL material.

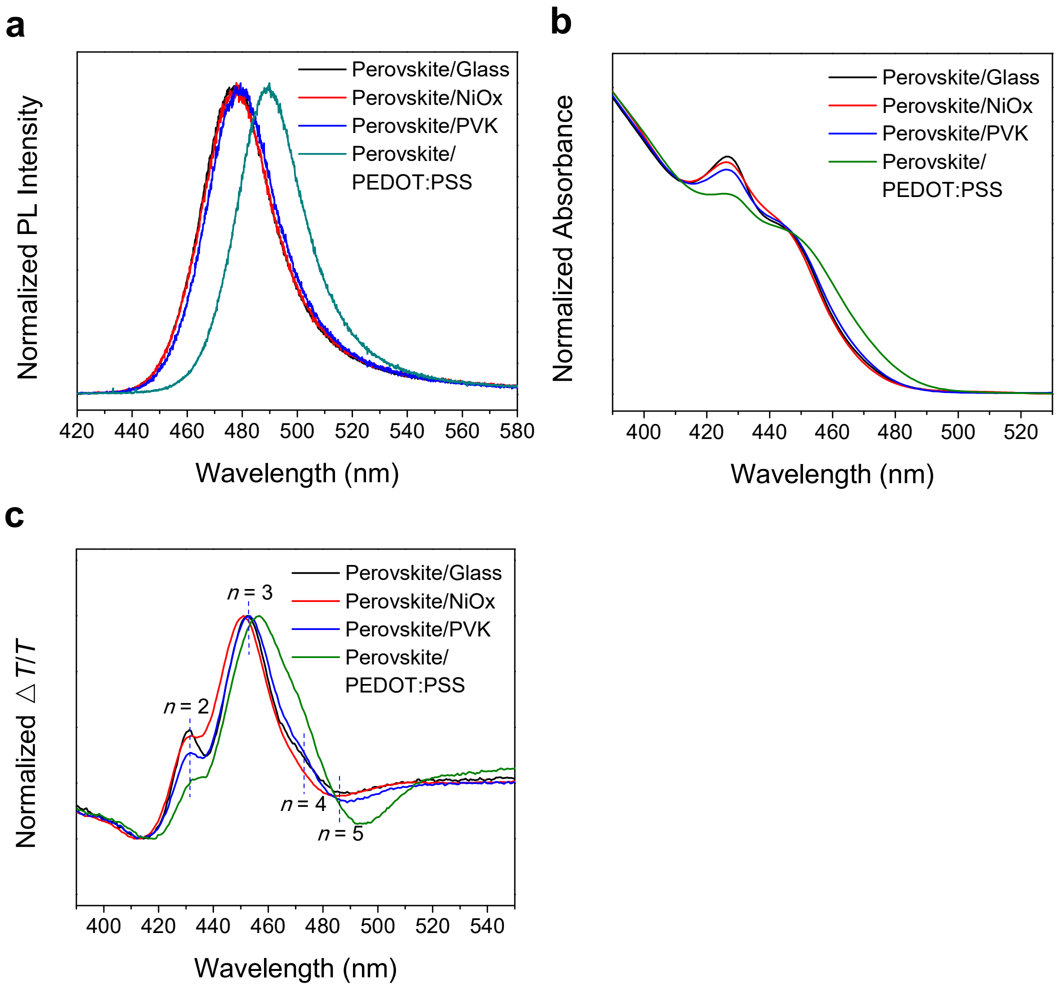

Supplementary Figure 9. (a) PL, (b) absorption and (c) TA spectra of perovskite films Sample V on glass, NiO_x, PVK, and PEDOT:PSS substrate.

Supplementary Table 1. Comparison of the performance of quasi-2D blue PeLEDs.

Compositions	EQE (%)	Luminance (cd m^{-2})	EL Peak Wavelength (nm)	PL Peak Wavelength (nm)	Ref
$POEA_2MA_{n-1}Pb_nBr_{3n+1}$	1.1	19.5	480, 494, 508	462, 480, 492, 508	ref[1]
	0.06	1.26	462	464, 480	
$OLA_2MA_{n-1}Pb_nBr_{3n+1}$	0.004	1	432, 480	436	ref[2]
$BA_2MA_{n-1}Pb_nBr_{3n+1}$	0.0054	~1	~440, 460, 475	~410, 440, 490	ref[3]
$4\text{-}PBA_2Cs_{n-1}Pb_nBr_{3n+1}$	0.015	186	435, 466, 491	435, 466, 475	ref[4]
$EA_2MA_{n-1}Pb_nBr_{3n+1}$	2.6	200	473, 485	473, 485	ref[5]
$IPA/PEA_2MA/Cs_{n-1}Pb_nBr_{3n-1}$	1.5	**2480**	490	488	this work

POEA: 2-phenoxyethylammonium

OLA: oleylammonium

BA: n-butylammonium

4-PBA: 4-phenylbutylammonium

EA: ethylammonium

MA: methylammonium

Cs: cesium

Supplementary Note 1.

According to the formula $PEA_2A_{n-1}Pb_nBr_{3n+1}$ (A = Cs, MA), we could prepare the precursor solution with the following molar ratio.

1. 2PEABr + ABr + 2PbBr$_2$ (*n* = 2)

If 50% PEA is used to form phase $n = 1$ firstly, residual composition is

PEABr + ABr + 1.5PbBr$_2$ (***n* = 3**)

If 75% PEA is used to form phase $n = 1$ firstly, residual composition is

0.5PEABr + ABr + 1.25PbBr$_2$ (***n* = 5**)

If 80% PEA is used to form phase $n = 1$ firstly, residual composition is

0.4PEABr + ABr + 1.2PbBr$_2$ (***n* = 6**)

…

2. 2PEABr + 2ABr + 3PbBr$_2$ (*n* = 3)

If 50% PEA is used to form phase $n = 1$ firstly, residual composition is

PEABr + 2ABr + 2.5PbBr$_2$ (***n* = 5**)

…

3. 2PEABr + 4ABr + 5PbBr$_2$ (*n* = 5)

If 50% PEA is used to form phase $n = 1$ firstly, residual composition is

PEABr + ABr + 4.5PbBr$_2$ (***n* = 9**)

…

Supplementary References

1 Chen, Z. *et al.* High-performance color-tunable perovskite light emitting devices through structural modulation from bulk to layered film. *Adv. Mater.* **29**, 1603157 (2017).

2 Kumar, S. *et al.* Efficient blue electroluminescence using quantum-confined two-dimensional perovskites. *ACS Nano* **10**, 9720–9729 (2016).

3 Congreve, D. N. *et al.* Tunable light-emitting diodes utilizing quantum-confined layered perovskite emitters. *ACS Photonics* **4**, 476-481 (2017).

4 Cheng, L. *et al.* Sky-blue perovskite light-emitting diodes based on quasi-two-dimensional layered perovskites. *Chin. Chem. Lett.* **28**, 29-31 (2017).

5 Wang, Q., Ren, J., Peng, X.-F., Ji, X.-X. & Yang, X.-H. Efficient sky-blue perovskite light-emitting devices based on ethylammonium bromide induced layered perovskites. *ACS Appl. Mater. Interfaces* **9**, 29901–29906 (2017).
